# Density of *Dermacentor reticulatus* Ticks in Eastern Poland

**DOI:** 10.3390/ijerph17082814

**Published:** 2020-04-19

**Authors:** Zbigniew Zając, Aneta Woźniak, Joanna Kulisz

**Affiliations:** Chair and Department of Biology and Parasitology, Medical University of Lublin, 20-080 Lublin, Poland; aneta.wozniak@umlub.pl (A.W.); joanna.kulisz@umlub.pl (J.K.)

**Keywords:** tick density, *Dermacentor reticulatus*, tick distribution, tick occurrence

## Abstract

*Dermacentor reticulatus*, the ornate cow tick, is second only to *Ixodes ricinus* as the most important reservoir and vector of infectious diseases in Europe. In recent years, the distribution of *D. reticulatus* ticks has expanded into new territories, including increased population densities in areas of their previous occurrence. Our investigations around this consisted of two stages. In the first stage, we monitored the seasonal activity of *D. reticulatus* ticks in Polesie National Park in 2014–2019. The second stage, which was carried out in 2019 at the peak of the spring (March) and autumn (October) activity of this species, included assessment of the density of *D. reticulatus* ticks in the entire province. To this end, the study area was divided into 101 equal plots that were surveyed for ticks. The seasonal activity of *D. reticulatus* in Polesie National Park showed peaks of activity in autumn in 2014–2018 and in spring in 2019. A total of 19,559 adult *D. reticulatus* specimens were collected, with a mean of 96.8 specimens/100 m^2^ in Lublin Province. The area of Lublin Province is characterized by a high density of the ornate cow tick. An increase in the surface area of meadows and fallow land has contributed to a rise in the number of local populations of *D. reticulatus* ticks.

## 1. Introduction

*Dermacentor reticulatus*, the ornate cow tick, is one of the most widely distributed tick species throughout Europe. As suggested by some authors, two geographically separated populations of ornate cow ticks, known as the “Eastern European” and “Western European”, can be distinguished in Europe. This phenomenon is not found in other widely distributed tick species in Europe, such as *Ixodes ricinus* or *D. marginatus* [1,2,3].

In the western part of the continent, the occurrence of *D. reticulatus* has been confirmed in the northern regions of Spain, France, the southern part of the British Isles, Switzerland, Benelux countries, and Germany [3,4,5]. In Central Europe, numerous localities of this species have been reported in Poland, Hungary, the Slovakian lowlands, Slovenia, and Austria [3,6,7,8,9]. In the east, the occurrence range of *D. reticulatus* ticks covers Ukraine, Belarus, Lithuania, Latvia, Estonia, and the European north-west part of Russia. Numerous records have also been reported from the Caucasus and Asia to the Omsk and Novosibirsk regions [2,10,11,12,13].

Before the beginning of the 21st century, it was thought that the distribution of *D. reticulatus* in Poland was limited to the territory east of the Vistula River and in the north-eastern part of the country, while western regions were considered non-endemic. Additionally, it was assumed that Poland was the boundary between the eastern and western populations of *D. reticulatus* [13,14,15].

Studies on tick ecology and distribution, which have intensified in recent years, have confirmed the occurrence of *D. reticulatus* in the northern, western, and south-western territories of Poland [16,17,18,19,20,21,22].

Similar changes in the dynamics and spread of *D. reticulatus* into new areas have also been observed in other European countries, including in Germany, where its recorded distribution before 1970 was limited to western areas, with low numbers reported [23]. In the following years, the distribution of *D. reticulatus* was reported to expand into eastern areas bordering Poland [6]. Many new sites of *D. reticulatus* occurrence have been discovered in the Czech Republic. In Slovakia, new localities of the ornate cow tick, representing both eastern and western European populations, have been reported [24,25]. More recently, *D. reticulatus* ticks have been reported in Hungary [26,27,28], where only two sites were documented before the 1970s [9]. Increased abundance and new localities of *D. reticulatus* have also been observed in Romania [29,30]. Clustered populations of *D. reticulatus* have been confirmed in the Balkans [31,32,33,34] and Iberian Peninsula [35]. The spread of ornate cow ticks to Northern Italy [36], which was previously regarded as tick-free, has been observed recently.

*D. reticulatus* populations in Europe were previously separated by a geographical border, which is now breaking down due to new introductions. Although genetic studies of specimens of both populations have shown differences in the structure of their genomes [37], the results of these studies do not indicate equivocally the cause of the occurrence of gaps in *D. reticulatus* distribution in Europe. 

The aim of this study was to conduct concurrent large-scale tick surveillance to determine the relative density and distribution of *D. reticulatus* in Lublin Province, Eastern Poland.

## 2. Materials and Methods

The investigations consisted of two stages. In the first stage (2014–2019), we monitored the seasonal activity of *D. reticulatus* ticks in a habitat preferred by the species in Polesie National Park. The results facilitated precise determination of the rhythms of seasonal activity of the species in the spring–autumn period between March and October. The second stage (2019) was focused on assessment of the density of *D. reticulatus* ticks in the entire Lublin Province.

### 2.1. Study Area

#### 2.1.1. Polesie National Park

Polesie National Park (51.496° N, 23.102° E) is located in the lowlands of Central-Eastern Poland. The park, covering a surface area of 97.6 km^2^, is mainly occupied by lakes, ponds, marshes, peat bogs, and meadows [38].

#### 2.1.2. Lublin Province

**Figure 1:**
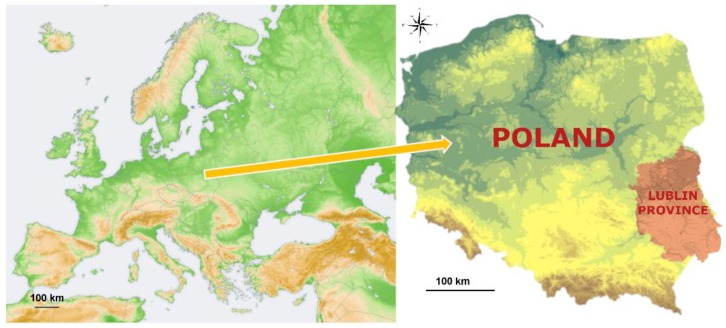
Maps of the study area in Eastern Poland, based on Wikimedia [39,40] with our own modifications.

The Province has an area of 25,122 km^2^, which accounts for 8.02% of the territory of Poland (Figure 1).

**Figure 2:**
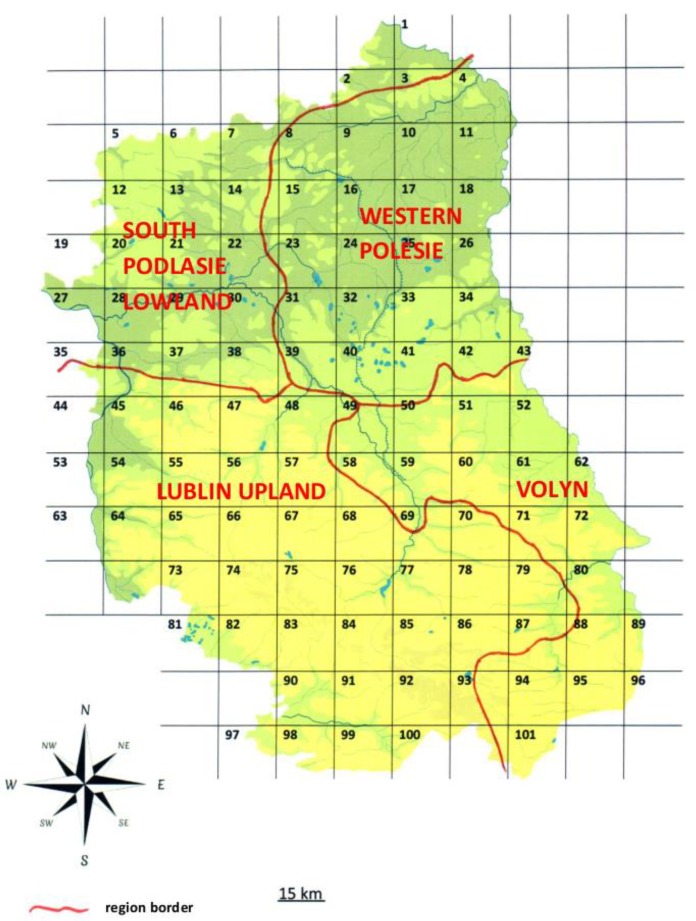
Division of Lublin Province into study plots and physico-geographical regions of Lublin Province, based on Kondracki [41] and Wikimedia [42], with our own modifications.

Lublin Province is located in an area of four macroregions (Western Polesie, Volyn, South Podlasie Lowland, and Lublin Upland) differing in geomorphological structure, water features, and climate (Figure 2) [41].

### 2.2. Tick Collection Sites

#### 2.2.1. Polesie National Park

**Figure 3:**
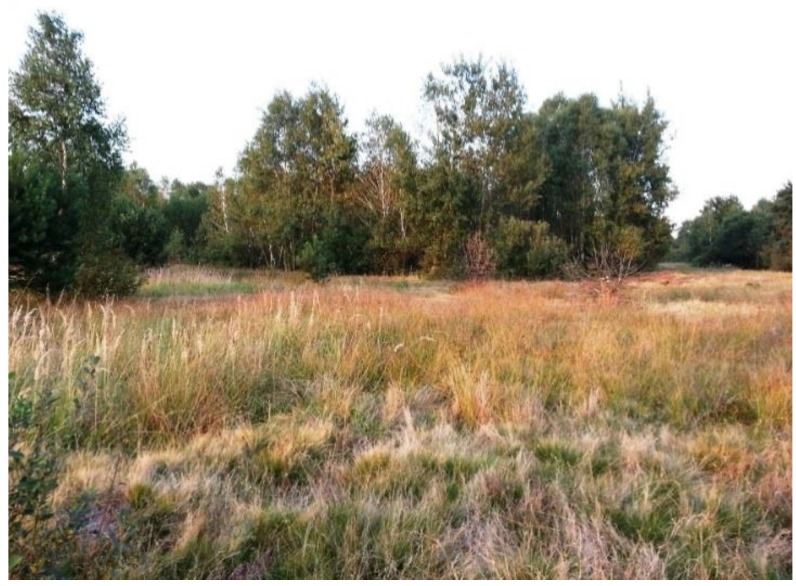
*Dermacentor reticulatus* collection site in Polesie National Park.

*D. reticulatus* ticks were collected in a habitat preferred by this species in Polesie National Park [43] (Figure 3).

#### 2.2.2. Lublin Province

A grid with sides corresponding to 15.8 km of terrain was applied to the study site, resulting in 101 squares with a mean area of 250 km^2^ each, representing 1% of the landmass of Lublin Province (Figure 2).

**Figure 4:**
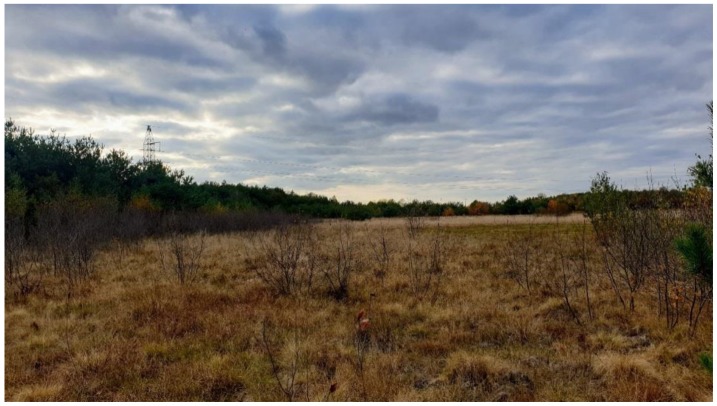
Site of tick collection, plot 17.

Next, satellite photos available in Google Maps were used to establish a potential tick collection site in each square. Grasslands undergoing ecological succession located at the margins of or in close vicinity to forests, and with access to watercourses, were regarded as habitats preferred by these ticks [43]. In each square of the grid, one potential approximately 2500 m^2^ site of tick collection was established. The next step involved visual inspection of the previously established tick collection sites. In sites that met the assumed criteria, one 100 m^2^ plot was delineated (Figure 4) for subsequent collection of ticks. In the event of discrepancies between the satellite images of the area and the actual situation, the procedure of selection of the sites was repeated (the selection of plots 34, 55, 62, and 75 was repeated once).

### 2.3. Tick Surveillance

*D. reticulatus* ticks were collected in Polesie National Park in 2014–2019. In 2014–2018, the ticks were collected at equal 14–20 day intervals. Month-long intervals were used for collection of ticks in 2019. Each time, the ticks were collected between 11:00 and 14:00 with the flagging method, in accordance with the procedure described by Nowak-Chmura [44]. Throughout the study period in Polesie National Park, the ticks were collected by the same person. The vegetation was swept with a 1 m^2^ white sheet each time for 30 min. A 100 m long and 1 m wide transect was covered, which corresponds approximately to 100 m^2^. The sheet was inspected approximately every 2 min and the attached ticks were removed using forceps, placed in a container, and transported to the central laboratory. 

**Figure 5:**
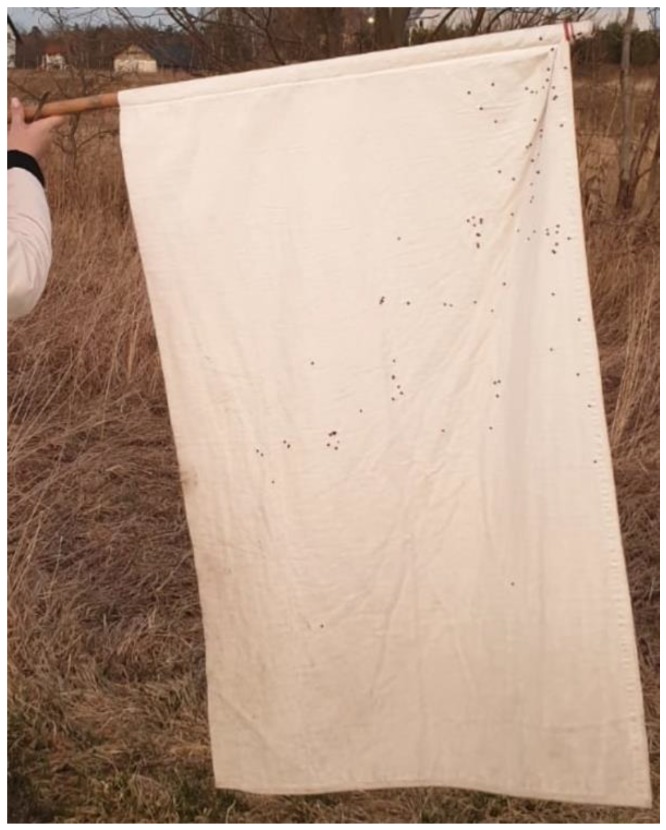
*Dermacentor reticulatus* ticks collected in plot 41. A total of 96 adult specimens were collected in an area of 10 m^2^**.**

The investigations of the tick density in Lublin Province were conducted during seasonal peaks of tick activity in this region indicated in the literature [43,45,46], based on the results of studies of the seasonal activity of *D. reticulatus* ticks in Polesie National Park (Table A1 in Appendix A) and concurrent tick surveillance of the *D. reticulatus* activity in different areas of Lublin Province. The tick surveillance was conducted in the spring from 18 to 31 March 2019, and from 12 to 24 October 2019 in the autumn. Plot 93 was excluded during the autumn due to ongoing construction. Ticks were collected with the standard flagging method. Vegetation was swept with 1 m^2^ white flannel fabric. Each time, the ticks were collected from a 100 m^2^ plot. This area was flagged over a distance of 10 m and then the flag was checked for the presence of ticks on both sides (Figure 5). This was repeated 10 times to ensure that the entire surface area had been swept. The ticks were collected once in the spring and once in the autumn, each time from exactly the same 100 m^2^ area.

In both stages, the current weather conditions, temperature, and relative air humidity were measured using Data Logger R6030 (Reed Instruments, Wilmington, NC, USA). In the laboratory, the species and sex of collected specimens were identified with the use of a stereoscopic microscope Zeiss STEMI DV4 (Carl Zeiss Light Microscopy, Göttingen, Germany) and an identification guide compiled by Estrada-Peña et al. [47]. 

### 2.4. Analysis of the Land Structure of the Study Area

The Google Maps and a layer of satellite images of the region [48] were used for calculation of the land use structure in each plot of the superimposed cartographic grid. The area of cultivated fields (arable land), forests, and meadows (grasslands) was calculated in this way. The designation “other” referred to buildings, water reservoirs, and areas that could not be clearly defined.

### 2.5. Statistical Analysis

Differences in the number of active *D. reticulatus* females and males in each experimental plot were analyzed statistically using the Wilcoxon signed-rank test. The Kruskal–Wallis test was used for statistical analysis of the number of active *D. reticulatus* females and males in the spring and autumn periods.

A multiple regression model and Spearman’s rank correlation tests were used to assess the impact of the land use on tick densities. The effect of temperature and relative humidity on tick activity in Polesie National Park was evaluated using the Kruskal–Wallis test.

The statistical analysis was conducted using Statistica 10PL (StatSoft, TIBCO Software Inc, Palo Alto, CA, USA) software, and the significance level for all statistical tests was *p* < 0.05.

## 3. Results

### 3.1. Multiannual Monitoring of the Seasonal Activity of D. reticulatus Ticks

In Polesie National Park, a statistically significant (H = 6.98, *p* = 0.047) tendency towards increased numbers of active *D. reticulatus* was observed every year in 2014–2018. Higher numbers of active ticks were reported in the autumn. Concurrently, higher numbers of females than males were collected. In 2019, there were changes in the seasonal activity of *D. reticulatus*, as higher numbers of active ticks were collected in the spring (on average 165.5 individuals per collection event: 97 females and 68.5 males) than in the autumn (on average 46.5 individuals per collection event: 28.5 females and 18 males) (Table 1). Throughout the study, the seasonal activity (spring–autumn) of *D. reticulatus* ticks was significantly influenced by the air temperature prevailing during tick collection (H = 12.887, *p* = 0.002).

### 3.2. Density of D. reticulatus Ticks

A total of 19,559 adult (11,598 females and 7961 males) *D. reticulatus* were collected throughout the study period (Table A2). The mean number of ticks collected in Lublin Province was 96.8 specimens/100 m^2^, with a significant dominance of females (on average 57.6 females and 39.2 males/100 m^2^) (Z = 8.19, *p* < 0.001). The adult tick population densities were higher in the autumn than in the spring (Table 2, Table A2).

**Figure 6:**
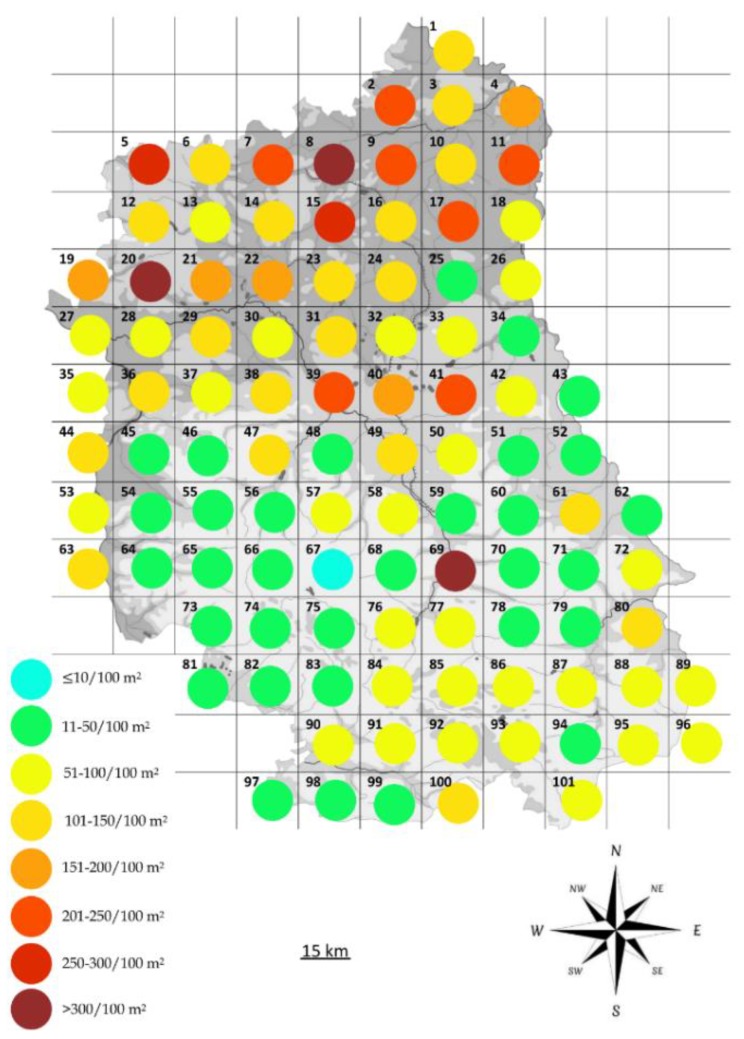
Spatial distribution of the average density of *D. reticulatus* populations in Lublin Province, the map outline based on Wikimedia [42] with our own modifications.

While the density of *D. reticulatus* in Lublin Province was high, the mean numbers were unevenly distributed (Figure 6). The highest numbers of ticks were collected in the northern part of the study area (plots 1–18; South Podlasie and Western Polesie), which varied from 86 specimens with a clear dominance of females over males (on average 53 females and 33 males) to 311 specimens/100 m^2^ (224 females and 86.5 males). The highest density of *D. reticulatus* ticks throughout the study period was observed in plot 69, located in Lublin Upland (Figure 4). During a single collection event at the spring activity peak, 516 adults/100 m^2^ of the ornate cow tick were collected, including 297 females and 219 males (Figure 6, Table A2). Low mean numbers of adults, ranging from 10 to 100 specimens/100 m^2^, were collected from nearby sites (located in the same macro-region) (Figure 6, Table A2). The lowest number of adult *D. reticulatus* was collected in the central part of the province, in plot 67, with 5 ticks/100 m^2^ (with an average of 3 females and 2.5 males) (Figure 6, Table A2).

At the peak of the seasonal (spring and autumn) activity of *D. reticulatus* ticks in Lublin Province, there were differences in the distribution of tick density. The northern, southern, and western parts of the Lublin region were characterized by a higher density of *D. reticulatus* ticks in the autumn, while a higher density in the spring was noted in the central and eastern parts of the area (Table A2, Figure 6).

The density of the ornate cow tick based on ecological variables (arable fields, forests, meadows) was not significantly different (F_(3.97)_ = 1.05, *p* = 0.3728). However, the analysis of Spearman’s rank correlation coefficient indicated that the density of *D. reticulatus* (both females and males) rose with increased meadow area (Rs = 0.281, *p* = 0.0044). 

## 4. Discussion

Population densities of *D. reticulatus* are dependent upon biotic and abiotic factors. The most important factors include the presence of potential hosts, ecological habitats, climatic factors, photoperiod, excreted semiochemical substances, and hormones controlling the rhythms of seasonal activity, diapause periods, and biology of tick reproduction and development [12,49].

In Eastern Poland, the greatest impact on the seasonal (spring–autumn) and diurnal rhythms of *D. reticulatus* activity is exerted by air temperatures prevailing during tick collection [43,45]. This dependence is supported by the results of multiyear monitoring of seasonal tick activity in Polesie National Park. Together with relative humidity, it can affect the ticks’ questing behavior [50] and locomotor activity [51].

The area of Lublin Province has the highest density of tick populations (mean 96.8 specimens/100 m^2^) of all studied areas in Poland. The mean number of adult *D. reticulatus* collected in Lublin Province was 13.1-fold higher than in Mazovia (Central Poland; mean 7.4 ticks/100 m^2^ from 2012–2014), 15.6-fold higher than in Lubuskie Province (Western Poland; mean 6.29 ticks/100 m^2^ from 2012–2014), and 32.3-fold higher than in the West Pomeranian Province (North-Western Poland; mean 3.04 ticks/100 m^2^ from 2012–2014) [20].

The northern and northeastern regions of Lublin Province are characterized by a high density of *D. reticulatus* ticks (Figure 6, Table A2). These areas have the largest proportion of grasslands in the entire province, and an increase in their surface area correlates positively and has a significant statistical effect on the number of collected ticks. A significant percentage of land in this area is also occupied by fallows and wasteland, as well as forest islands. The mosaic character of the landscape offers favorable conditions for the development of *D. reticulatus* populations. Forest areas provide availability of hosts of adult stages, whereas meadows and wasteland are preferable habitats for rodents, which are the hosts of juvenile ticks.

The highest island density of *D. reticulatus* ticks (516 specimens/100 m^2^) was observed in plot 69, located in the central part of the region (Figure 6). Of note, the tick densities in the neighboring sites (located in the same macro-region) were among the lowest in the entire study area, and ranged from 10 to 57 specimens/100 m^2^ (Figure 6, Table A2). Such a high island density of ornate cow ticks is associated with the ecology of the habitats. The site where the maximum number of ticks was caught is surrounded by an abandoned meadow, watercourse, and forest in close proximity, while the forest cover in the entire macro-region is <8% (average for the province: 22%) (Table A2). 

As demonstrated by Mierzejewska et al. [52], the spread of *D. reticulatus* is associated with the loss of forest area. We share this opinion, with the provision that excessive tree felling does not result in conversion of the land into intensively used agricultural areas. In the study area, the lowest average density of ornate cow ticks was observed in Lublin Upland, a region that is intensively used for agriculture due to its fertile soils. The impact of large forest areas on the density of ornate cow ticks is observed in Lublin Province as well. The number of ticks collected in plots 73 and 74 (forest cover >61%, meadow area 1.93%–4.09%), located within the biggest forest of Lublin Province (“Janów Forest”), was significantly lower than the average number for the region of 29 and 23 specimens/100 m^2^ in spring and 46 and 30 specimens/100 m^2^ in autumn, respectively (Table A2). The results of our studies show that *D. reticulatus* populations can persist and steadily develop only in areas with appropriate proportions of forests and meadows/fallow lands. Nevertheless, isolated populations of *D. reticulatus* ticks can exist in habitats where these proportions are disturbed.

*D. reticulatus* ticks mostly exhibit two distinct peaks of seasonal activity in spring and autumn. In the French Alps, the European part of Russia, and North-Eastern Poland, more ticks are active in spring [12,53,54]. The data published so far indicate that the number of ticks collected in autumn in Eastern Poland is three-fold higher than in spring [43,45,46]. Our research results show that the seasonal activity may change within the same population, even if it is recognized as stable (Table 1). The *D. reticulatus* population in Polesie National Park, monitored by us for the last 6 years (Table 1), exhibited a clearly higher number of active ticks in autumn in 2014–2018 and dominance of the spring peak of activity over autumn in 2019, with a ratio of 3.5:1 (Table 1). Since the experimental field is located within a protected area, the anthropogenic impact is limited. We believe that the size and dynamics of the *D. reticulatus* seasonal activity are influenced mostly by the availability of preferred hosts occurring in the investigated area, such as roe deer (*Capreolus capreolus*), deer (*Cervus elaphus*), elk (*Alces alces*), and wild boar (*Sus scrofa*). Our unpublished observations demonstrate the highest degree of ornate cow tick infestations in wild boars. These animals occur in the entire province and their population size has been growing over recent years, by as much as 178%, or from 6400 to 17,800 specimens within 5 years. For comparison, there were 2300 elks and 7600 deer in the area at the same time [55]. 

In 2017–2018, a decrease in the size of wild boar populations was observed in Lublin Province due to confirmed outbreaks of African swine fever (ASF). Before April 2018, the mortality caused by this viral disease was 1942 wild boars in Eastern Poland [56]. Furthermore, during this period, the wild boar population was reduced preventively. At the turn of 2018/2019, the estimated size of the wild boar population in the Province declined to “no more than several hundred individuals” (personal communication with the Lublin Hunting Association). This led to a significant decrease in the number of animals that are one of the main hosts of *D. reticulatus* [57,58]. In our opinion, this situation is reflected in the structure of the population size and the dynamics of seasonal activity of *D. reticulatus* populations in Eastern Poland. It also explains the differences in the spatial distribution of the density of this tick species in Lublin Province. Considering the over 2-year survival of adult specimens in a habitat [12], the predominance of tick activity in spring in areas where an autumn peak was noted previously [43] could be associated with insufficient numbers of hosts for the adult ornate cow ticks in this period. Nevertheless, impacts from other factors on this phenomenon should not be excluded, and further observations and investigations are required.

The high density of *D. reticulatus* ticks in Lublin Province is associated with a greater risk of tick attack on animals, and sporadically on humans, than in other country regions, and therefore an increased threat of transmission of tick-borne pathogens. Various pathogens have been detected in ornate cow ticks, including *Anaplasma phagocytophilum*, *Rickettsia raoultii*, *Borrelia burgdorferi* s. l., *Babesia* spp., and TBE virus [59,60]. An increase in the tick population density may result in an increased incidence of tick-borne diseases in future [61]. Numerous cases of canine babesiosis and domestic animal borreliosis have already been reported [62,63,64].

## 5. Conclusions

*D. reticulatus* ticks occur throughout the entire area of Lublin Province. This region is characterized by a very high density of the ornate cow tick. However, it is unevenly distributed. Changes in land use may influence the size of local *D. reticulatus* populations. An increase in the surface of meadows, grasslands, and fallow land contribute to a rise in the number of ornate cow ticks, whereas afforestation of new areas may reduce their population size.

The local *D. reticulatus* population exhibits differences in its peaks of seasonal activity. The highest risk of *D. reticulatus* infestations of animals and humans occurs in spring in the eastern and central parts of the region, and in autumn in the northern and western parts of the province.

## Figures and Tables

**Table 1:** Mean values of *D. reticulatus* ticks collected in Polesie National Park per 100 m^2^.

Year	Spring						Autumn					
	*n*	T [°C]	H [%]	F	M	F+M	*n*	T [°C]	H [%]	F	M	F+M
2014	6	11.8	77.2	10.3	6.8	17.1	3	23.0	72.2	26.6	22.7	49.3
2015	7	14.9	69.3	11.1	7.0	18.1	4	20.8	76.3	21.0	19.2	40.2
2016	6	12.8	81.0	10.2	8.3	18.5	4	18.1	68.2	27.0	17.5	44.5
2017	6	13.4	77.2	13.8	13.2	27.0	5	17.8	75.4	25.6	19.4	45.0
2018	3	12.7	79.0	41.3	37.0	78.3	2	21.0	68.0	77.5	60.0	137.0
2019	2	13.5	80.2	97	68.5	165.5	2	19.8	74.6	28.5	18	46.5

*n*—number of collection events, T—mean temperature during the collection event, H—mean relative air humidity during the collection event, F—females, M—Males.

**Table 2:** Mean density of *D. reticulatus* ticks per 100 m^2^ in Lublin Province.

Study Period	Sex	*n*	Mean Numbers	SD	Median	Min	Max
Spring	F	101	56.5	50.6	41.0	4.0	297.0
	M	101	38.4	34.8	25.0	1.0	219.0
	F+M	101	94.9	83.4	68.0	5.0	516.0
Autumn	F	100	58.9	51.3	41.0	2.0	248.0
	M	100	40.9	33.5	30.0	1.0	135.0
	F+M	100	98.9	83.3	67.0	0.0	370.0
Whole study period	F	101	57.5	44.4	43.5	3.0	224.5
	M	101	39.5	28.3	31.5	2.5	128.5
	F+M	101	96.8	71.7	73.5	5.5	311.0

SD—standard deviation, *n*—number of plots, F—females, M—males, Min—minimum, Max—Maximum.

## Appendix A

**Table A1:** Seasonal activity of *D. reticulatus* ticks in Polesie National Park in 2014–2019 per 100 m^2^.

Collection Date	F	M	F+M
23 March 2014	15	10	25
8 April 2014	15	5	20
23 April 2014	8	5	13
1 May 2014	10	8	18
10 May 2014	10	6	16
20 May 2014	4	7	11
17 September 2014	18	18	36
15 October 2014	29	30	59
29 October 2014	33	20	53
21 March 2015	18	15	33
1 April 2015	15	10	25
11 April 2015	13	8	21
25 April 2015	17	3	20
1 May 2015	5	8	13
23 May 2015	5	5	10
5 June 2015	5	0	5
12 September 2015	22	24	46
26 September 2015	20	18	38
12 October 2015	30	19	49
2 November 2015	12	16	28
5 March 2016	13	10	23
4 April 2016	8	12	20
16. April 2016	12	12	20
30 April 2016	10	8	18
7 May 2016	8	3	11
3 June 2016	10	5	15
9 September 2016	22	11	33
20 September 2016	17	14	31
30 September 2016	30	25	55
20 October 2016	39	20	59
10 March 2017	8	12	20
20 March 2017	28	20	48
1 April 2017	15	21	36
19 April 2017	6	8	14
25 April 2017	13	10	23
10 May 2017	13	8	21
5 September 2017	14	10	24
20 September 2017	30	33	63
27 September 2017	25	26	51
11 October 2017	20	8	28
18 October 2017	39	20	59
12 March 2018	31	35	66
24 March 2018	62	35	97
30 March 2018	31	41	72
19 September 2018	61	29	90
19 September 2018	94	91	185
1 March 2019	55	70	125
28 March 2019	139	67	206
19 September 2019	11	17	28
10 October 2019	46	19	65

F—females, M—males.

**Table A2:** Structure of land use and density of *D. reticulatus* ticks per 100 m^2^ in the study area.

Plot Number/Regions	Landscape Structure				Ticks Density Per 100 m^2^												
					Spring					Autumn					Total Study Period		
	Farmlands [%]	Forests [%]	Meadows [%]	Other [%]	Weather Parameters		Tick Density			Weather Parameters		Tick Density			Mean Tick Density		
					T [°C]	H [%]	F	M	F+M	T [°C]	H [%]	F	M	F+M	F	M	F+M
1	67.21	13.5	5.21	14.08	12.1	88.8	84	54	138	22.5	60.0	98	61	159	91	57.5	148.5
2	68.00	20.65	5.98	5.37	10.8	87.0	105	75	180	21.8	70.6	150	122	272	127.5	98.5	226
3	61.00	15.59	5.23	18.18	13.4	90.6	29	14	43	23.2	70.1	100	69	169	64.5	41.5	106
4	55.11	29.96	7.99	6.94	13.5	80.5	74	51	125	23.1	70.6	112	82	194	93	66.5	159.5
5	79.56	5.66	2.05	12.73	14.1	86.0	153	93	246	19.6	81.0	177	106	283	165	99.5	264.5
6	65.12	18.20	6.78	9.90	15.1	75.4	45	25	70	20.5	67.5	130	96	226	87.5	60.5	148
7	80.12	10.10	4.11	5.67	14.8	81.2	105	66	171	22.5	66.0	164	102	266	134.5	84	218.5
8	64.99	18.70	6.78	9.53	14.7	70.0	201	51	252	23.4	72.5	248	122	370	224.5	86.5	311
9	40.00	45.03	6.50	8.47	17.5	77.0	96	48	144	21.0	77.8	190	134	324	143	91	234
10	71.20	11.96	8.98	7.86	16.1	86.0	44	30	74	19.8	69.0	116	81	197	80	55.5	135.5
11	55.10	30.11	8.21	6.58	14.5	70.5	93	93	186	20.0	54.2	170	110	280	131.5	101.5	233
12	73.30	16.41	6.10	4.19	12.9	77.9	26	19	45	18.5	70.5	145	98	243	85.5	58.5	144
13	85.23	4.29	2.70	7.78	15.0	84.2	31	36	67	17.5	81.2	56	36	92	43.5	36	79.5
14	77.40	13.04	1.80	7.76	12.1	81.8	75	39	114	16.1	84.2	88	24	112	81.5	31.5	113
15	83.90	3.61	6.67	5.82	13.7	74.0	123	87	210	15.1	75.9	196	108	304	159.5	97.5	257
16	65.21	18.47	7.45	8.87	11.9	86.1	84	42	126	13.9	77.7	99	55	154	91.5	48.5	140
17	60.21	25.00	7.00	7.79	15.6	82.0	102	81	183	18.8	78.3	190	100	290	146	90.5	236.5
18	55.12	30.56	6.12	8.20	16.1	76.0	42	35	77	20.5	71.0	64	31	95	53	33	86
19	65.12	24.87	3.15	6.86	18.5	67.0	68	56	124	16.9	79.9	133	70	203	100.5	63	163.5
20	65.18	17.30	7.87	9.65	22.0	66.0	166	122	288	21.0	65.7	180	135	315	173	128.5	301.5
21	80.99	6.68	2.10	10.23	19.5	69.9	177	69	246	23.0	69.0	75	50	125	126	59.5	185.5
22	70.36	21.67	3.98	3.99	15.4	78.9	129	99	228	22.0	54.9	60	64	124	94.5	81.5	176
23	71.33	18.90	5.00	4.77	16.7	69.2	100	65	165	19.8	55.0	62	41	103	81	53	134
24	60.12	25.53	7.00	7.35	14.8	67.0	141	75	216	17.5	87.0	36	18	54	88.5	46.5	135
25	45.00	39.56	7.11	8.33	10.5	90.5	15	13	28	16.4	80.0	20	50	70	17.5	31.5	49
26	70.01	12.24	3.98	13.77	11.0	82.0	45	39	84	17.0	81.0	26	13	39	35.5	26	61.5
27	50.98	32.11	3.00	13.91	19.3	82.8	22	11	33	13.6	88.0	66	22	88	44	16.5	60.5
28	70.11	17.53	6.18	6.18	18.5	71.6	49	22	71	17.4	80.2	66	50	116	57.5	36	93.5
29	70.96	11.72	5.23	12.09	17.1	75.9	75	35	110	23.1	65.0	59	66	125	67	50.5	117.5
30	55.00	25.84	8.01	13.15	16.5	65.0	45	25	70	22.0	53.2	58	44	102	51.5	34.5	86
31	40.19	43.67	5.00	11.14	10.7	83.0	61	50	111	24.8	55.0	84	68	152	72.5	59	131.5
32	71.11	18.31	5.45	5.13	10.9	81.0	40	34	74	25.6	45.7	37	10	47	38.5	17	55.5
33	35.23	56.19	4.00	4.58	13.5	73.5	25	18	43	24.0	58.0	62	35	97	43.5	26.5	70
34	14.18	72.00	9.12	4.70	13.0	79.0	18	11	29	20.9	74.8	15	5	20	16.5	8	24.5
35	69.27	19.45	5.00	6.28	14.0	65.8	26	17	43	14.5	80.0	30	32	62	28	24.5	52.5
36	78.16	8.00	7.12	6.72	13.7	58.9	69	96	165	15.6	69.0	60	58	118	64.5	77	141.5
37	80.50	2.03	5.90	11.57	11.0	70.0	58	60	118	20.5	73.3	34	26	60	46	43	89
38	80.10	9.06	4.17	6.67	9.5	79.0	144	81	225	22.0	60.0	14	12	26	79	46.5	125.5
39	68.00	16.29	5.22	4.49	12.5	78.0	181	98	279	23.0	62.4	84	66	150	132.5	82	214.5
40	60.05	25.24	7.01	7.70	13.1	85.0	120	111	231	21.0	55.8	58	30	88	89	70.5	159.5
41	65.19	14.06	8.08	12.67	13.0	68.9	194	137	331	22.1	67.0	57	36	93	125.5	86.5	212
42	50.00	40.00	5.23	4.77	14.9	60.1	80	60	140	20.5	73.8	15	6	21	47.5	33	80.5
43	30.12	60.00	4.00	5.88	12.0	68.3	44	22	66	23.5	73.0	16	22	38	30	22	52
44	61.87	23.32	2.55	12.26	14.7	67.0	63	66	129	16.9	83.8	85	80	165	74	73	147
45	80.99	9.26	2.99	6.76	13.0	78.0	35	18	53	18.0	80.6	4	12	16	19.5	15	34.5
46	90.01	1.35	1.81	6.83	11.0	77.0	10	8	18	23.5	75.5	32	20	52	21	14	35
47	50.09	6.01	5.00	38.90	9.1	85.0	46	36	82	25.7	49.0	90	61	151	68	48.5	116.5
48	58.98	29.05	4.90	7.07	8.5	88.0	28	18	46	23.0	57.0	20	26	46	24	22	46
49	70.76	13.03	6.98	9.23	12.0	80.0	82	60	142	21.9	69.8	80	71	151	81	65.5	146.5
50	76.32	13.50	4.12	6.06	12.9	83.5	44	40	84	22.0	74.0	16	11	27	30	25.5	55.5
51	64.95	18.73	4.76	11.56	12.6	81.1	30	18	48	17.0	87.0	10	9	19	20	13.5	33.5
52	65.85	18.31	5.98	9.86	13.0	78.8	21	12	33	12.9	88.0	12	7	19	16.5	9.5	26
53	75.66	13.53	1.90	8.91	10.8	90.0	26	9	35	15.6	76.0	74	77	151	50	43	93
54	76.98	13.04	1.40	8.58	12.0	81.5	18	24	42	19.9	73.1	32	20	52	25	22	47
55	80.01	6.03	1.00	12.96	15.9	75.0	12	16	28	17.8	72.7	29	12	41	20.5	14	34.5
56	82.03	6.20	3.09	8.68	17.5	74.1	36	20	56	17.0	60.6	10	2	12	23	11	34
57	79.06	8.30	5.99	6.65	14.0	81.0	56	48	104	13.0	90.0	14	9	23	35	28.5	63.5
58	71.11	13.06	6.89	8.94	18.3	70.0	40	22	62	11.9	93.2	28	12	40	34	17	51
59	70.20	15.74	6.34	7.72	16.4	65.3	18	16	34	15.7	85.2	16	22	38	17	19	36
60	40.56	39.62	8.00	11.82	17.5	53.8	35	23	58	20.1	74.8	7	11	18	21	17	38
61	74.58	10.24	6.12	9.06	15.0	67.0	78	75	153	20.0	67.0	25	29	54	51.5	52	103.5
62	50.75	35.65	4.00	9.60	14.8	74.9	22	10	32	23.1	63.0	25	17	42	23.5	13.5	37
63	80.00	8.16	2.98	8.86	10.5	80.3	45	12	57	19.5	76.9	84	76	160	64.5	44	108.5
64	71.00	18.01	3.09	7.90	11.0	86.0	36	45	81	19.0	71.0	12	8	20	24	26.5	50.5
65	65.07	15.63	5.01	14.29	9.1	80.0	15	10	25	23.3	46.0	11	6	17	13	8	21
66	89.88	1.85	1.03	7.24	17.4	70.0	14	7	21	18.7	69.4	12	11	23	13	9	22
67	80.09	6.71	2.00	11.20	21.4	58.8	4	1	5	16.6	78.0	2	4	6	3	2.5	5.5
68	87.33	3.32	1.33	8.02	22.0	54.7	26	8	34	20.4	71.0	26	24	50	26	16	42
69	70.45	17.77	2.03	9.75	17.0	55.0	297	219	516	21.1	72.8	68	34	102	182.5	126.5	309
70	69.79	15.01	5.12	10.08	16.0	70.7	33	7	40	22.2	77.1	30	31	61	31.5	19	50.5
71	70.56	18.76	2.34	8.34	16.5	70.0	18	15	33	20.6	68.5	29	31	60	23.5	23	46.5
72	44.87	42.16	6.00	6.97	15.3	78.3	60	54	114	25.0	62.2	45	22	67	52.5	38	90.5
73	10.00	85.00	1.93	3.07	19.5	75.0	16	8	24	19.0	70.5	26	20	46	21	14	35
74	29.09	61.00	4.09	5.82	18.0	65.8	15	14	29	18.7	76.0	18	12	30	16.5	13	29.5
75	75.00	7.45	1.31	16.24	18.0	51.2	12	11	23	16.5	79.1	19	6	25	15.5	8.5	24
76	80.34	10.86	3.05	5.75	16.5	55.5	42	16	58	20.2	78.5	36	10	46	39	13	52
77	70.91	15.23	5.09	8.77	14.7	69.0	52	37	89	20.5	68.0	23	2	25	37.5	19.5	57
78	75.03	7.80	6.99	10.18	17.7	64.7	7	6	13	22.8	70.0	41	31	72	24	18.5	42.5
79	79.00	7.54	6.28	7.18	18.5	70.5	17	6	23	25.6	71.4	28	25	53	22.5	15.5	38
80	83.00	5.67	4.00	7.33	20.8	70.0	66	60	126	24.0	72.8	88	46	134	77	53	130
81	5.21	82.12	2.00	10.67	21.0	61.0	21	9	30	20.0	70.0	18	12	30	19.5	10.5	30
82	20.09	70.10	3.65	6.16	22.0	73.2	33	12	45	21.9	65.2	24	10	34	28.5	11	39.5
83	45.67	40.87	4.01	9.45	19.0	65.0	9	13	22	25.5	73.6	26	24	50	17.5	18.5	36
84	38.87	53.00	3.01	5.12	22.3	76.8	54	40	94	23.0	77.0	30	23	53	42	31.5	73.5
85	59.91	18.97	7.98	13.14	16.5	80.0	41	14	55	19.0	78.9	52	78	130	46.5	46	92.5
86	75.67	10.00	6.22	8.11	17.9	62.3	11	7	18	23.0	72.3	61	50	111	36	28.5	64.5
87	67.99	7.00	8.88	16.13	15.4	62.2	19	6	25	21.2	70.4	54	55	109	36.5	30.5	67
88	69.11	6.18	18.90	5.81	14.1	77.8	34	15	49	19.0	80.5	41	19	60	37.5	17	54.5
89	15.43	2.33	58.95	23.29	19.6	59.0	29	14	43	24.0	70.0	44	30	74	36.5	22	58.5
90	21.09	61.01	5.00	12.90	21.0	70.0	48	54	102	23.0	67.0	10	1	11	29	27.5	56.5
91	40.99	48.15	4.87	5.99	19.0	66.6	61	55	116	21.0	79.0	12	30	42	36.5	42.5	79
92	49.23	37.11	3.69	9.97	17.5	70.2	38	30	68	17.8	74.2	58	47	105	48	38.5	86.5
93	67.12	15.27	7.21	10.40	16.0	79.5	20	10	30	-	-	-	-	0	20	10	15
94	65.00	6.88	10.00	18.12	14.2	77.8	32	24	56	19.5	77.0	29	27	56	30.5	25.5	56
95	18.99	1.19	38.00	41.82	13.3	77.1	46	33	79	23.1	70.9	58	40	98	52	36.5	88.5
96	8.09	1.27	70.07	20.57	15.1	80.5	18	20	38	22.0	65.3	36	22	58	27	21	48
97	65.00	18.45	4.00	12.55	16.0	80.1	9	7	16	19.6	62.1	20	14	34	14.5	10.5	25
98	78.99	11.35	2.54	7.12	13.0	73.0	14	10	24	22.0	70.0	16	19	35	15	14.5	29.5
99	18.98	70.00	3.10	7.92	11.9	79.5	14	20	34	18.8	69.3	28	6	34	21	13	34
100	45.05	37.09	8.14	9.72	17.0	77.7	40	29	69	22.8	66.8	88	63	151	64	46	110
101	57.21	30.12	6.00	6.67	16.7	73.6	34	16	50	23.9	75.5	82	50	132	58	33	91
South Podlasie Lowland	71.96	14.33	4.74	9.06	15.4	75.5	84.5	56	140.5	19.7	70.5	92	62	154	88	59	147.5
Wester Polesie	56.19	29.17	6.39	7.97	13.6	77.7	86	56	141.5	20.7	69.0	91	59	150	89	57	146
Lublin Upland	61.74	24.75	3.80	9.68	15.9	72.1	35	25	60	19.6	69.9	32	25	58	32	25	59
Volyn	57.12	15.76	14.54	12.57	15.4	72.6	38	28	66	20.4	74.5	38	28	66	38	28	66

F—females, M—males, T—temperature, H—humidity.
